# Les masques trompeurs de la bipolarité: étude de 100 cas

**DOI:** 10.11604/pamj.2015.21.313.5409

**Published:** 2015-08-28

**Authors:** Fadoua Oueriagli Nabih, Abdesslam Benali, Imane Adali, Fatiha Manoudi, Fatima Asri

**Affiliations:** 1 Service de Psychiatrie, Hôpital Militaire d'Instruction Avicenne, Marrakech, Maroc; 2 Equipe de Recherche sur la Santé Mentale, CHU Mohamed IV, Université Caddi Ayyad, Faculté de Médecine, Marrakech, Maroc

**Keywords:** Retard diagnostic, Trouble bipolaire, masques trompeurs, diagnostic delay, Bipolar disorder, deceptive mask

## Abstract

Le trouble bipolaire (TB) est une pathologie dont la prévalence est estimée à 1-2%. Le diagnostic précoce du trouble constitue un enjeu thérapeutique majeur. L'objectif de ce travail est de déterminer les différents diagnostiques attribués aux patients bipolaires avant de recevoir le diagnostic adéquat et de préciser le délai moyen du retard diagnostique. C'est une étude descriptive transversale portant sur 100 patients atteints de TB, inclus selon les critères du DSM V, qui ont été vus en consultation ou bien hospitalisés dans le service de psychiatrie de l'hôpital Militaire Avicenne de Marrakech, durant une période de deux ans. L’âge moyen des patients était de 29,5 ans avec une prédominance masculine (80%). 40% de nos patients ont reçu au début un autre diagnostic que celui du TB et le premier diagnostic retenu était celui de l’épisode dépressif majeur dans 36% des cas, suivi de l'accès psychotique aigu dans 28% des cas, la schizophrénie dans 16,8% et le trouble de personnalité dans 10,2%. Le délai moyen du retard diagnostic était de 64 mois. 50% des patients ayant reçu un autre diagnostic avaient consulté au moins un psychiatre et 60% des patients avaient été hospitalisés au moins une fois avant le diagnostic du TB. Les errances diagnostiques du TB sont bien établies, conduisant forcément à un retard de prise en charge adéquate. Ces données devraient alerter les psychiatres pour favoriser un meilleur dépistage de la manie et de l'hypomanie qui restent les éléments clé du diagnostic du TB.

## Introduction

Le trouble bipolaire(TB) est une pathologie fréquente, sa prévalence dans la population générale est de 1 à 2% [1]. Ce trouble survient généralement chez le sujet jeune entre 25 et 35 ans. Le diagnostic positif du trouble n'est pas toujours évident. En effet, certaines formes atypiques ou mineures de début de la maladie peuvent masquer le diagnostic [2] conduisant à des errances thérapeutiques avec un retard de prise en charge adéquate qui aura un retentissement délétère sur l’évolution de la maladie et sur la vie familiale et socioprofessionnelle du sujet [3]. Nous avons mené une étude descriptive transversale auprès de 100 patients bipolaires vus au service de psychiatrie de l'hôpital Militaire Avicenne de Marrakech dont l'objectif est de déterminer les différents diagnostiques attribués aux patients bipolaires avant de recevoir le diagnostic adéquat et de préciser le délai moyen du retard diagnostique.

## Méthodes

C'est une étude descriptive transversale portant sur un échantillon de 100 patients atteints de troubles bipolaires, inclus selon les critères de DSM V, qui ont été vus en consultation ou bien hospitalisés dans le service de psychiatrie de l'Hôpital Militaire Avicenne de Marrakech, durant une période de deux ans, entre janvier 2012 et janvier 2014. Les données épidémiologiques et cliniques ont été recueillies à l'aide d'un hétéro-questionnaire renseignant sur: les caractéristiques sociodémographiques des patients, les antécédents personnels et familiaux, les premiers diagnostiques attribués aux patients avant le diagnostic du TB, le délai moyen du retard diagnostique, le nombre d'hospitalisations et le nombre de psychiatres vus avant le diagnostic du TB. Le retard diagnostic dans notre étude correspond à la période de troubles non traités séparant le début de la maladie (les premiers signes apparus avant le diagnostic du trouble bipolaire) et le diagnostic confirmé par le traitement adéquat. Tous les patients ont été interviewés en période normothymique. Le diagnostic du TB a été posé selon les critères DSM V (Diagnostic and StatisticalManual of Mental Disorders, Fifth Edition). La saisie et l'analyse des résultats de l’étude ont été faites par épi info 10, on a utilisé la prévalence test, considéré comme significative si P < 0,005.

## Résultats

L’âge moyen des patients était de 29,5 ans (+/- 7,9) avec un minimum de 16 ans, un maximum de 55 ans, et prédominance de la tranche d’âge comprise entre 25 et 35 ans. Notre échantillon était constitué de 80% d'hommes et de 20% de femmes.60% des patients étaient célibataires, et La majorité d'entre eux (76%) avaient un niveau d'instruction bas. 74% des patients étaient des militaires de fonction. 65,7% des patients consommaient des toxiques (60% de dépendance au tabac, 15,3% de dépendance à l'alcool et 28,5% de dépendance au cannabis). Les antécédents personnels d'accès hypomaniaques étaient présents dans 40% des cas, ceux d’épisodes dépressifs majeurs dans 30% des cas. 20,6% des patients avaient fait au moins une tentative de suicide avant le diagnostic du TB. Les antécédents familiaux psychiatriques étaient retrouvés dans 56,4% des cas, à type de trouble de l'humeur dans 49% des cas et de schizophrénie dans 7,4%, La totalité de nos patients avaient le diagnostic d'un TB type I.

**Figure 1:**
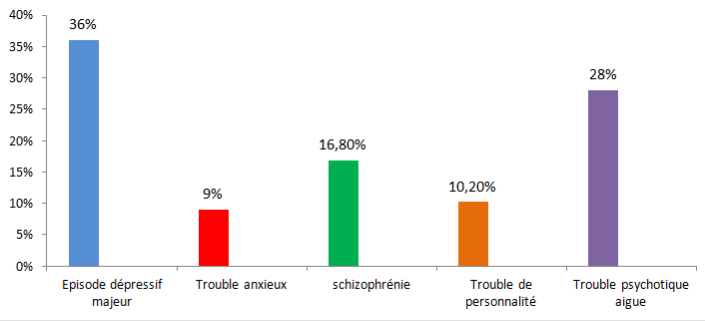
Répartition des premiers diagnostics retenus avant le diagnostic du troublebipolaire

**Figure 2:**
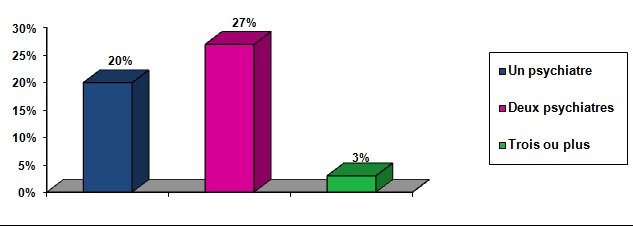
Nombre de psychiatres vus avant le diagnostic du trouble bipolaire

**Figure 3:**
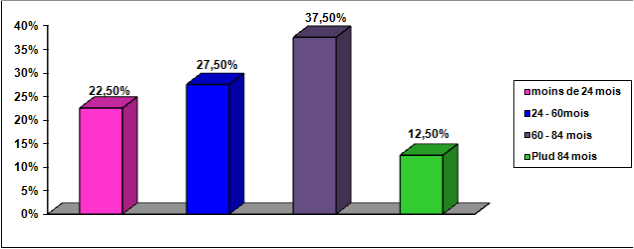
Durée moyenne du retard diagnostic du trouble bipolaire en mois

40% des patients ont reçu au début un autre diagnostic avant celui du TB. Le premier diagnostic retenu était un épisode dépressif majeur dans 36% des cas, accès psychotique aigu dans 28% des cas, une schizophrénie dans 16,8%, un trouble de personnalité dans 10,2% des cas et un trouble anxieux dans 9% des cas (Figure 1). La moitié des patients ayant reçu un autre diagnostic que celui du TB (20% de la totalité des patients inclus dans l’étude) avaient consulté au moins un psychiatre avant le diagnostic du TB (Figure 2) et 60% des patients ayant reçu un autre diagnostic que celui du TB (24% de la totalité des patients inclus dans l’étude) avaient été hospitalisé au moins une fois avant le diagnostic du TB. Le délai moyen de retard diagnostic était de 64 mois, +/- 33,45 avec un délai maximum de 132 mois. La moitié des patients avait un retard de plus de 60 mois (Figure 3).

## Discussion

Les erreurs diagnostiques du TB ont été objectivées par plusieurs études [4–6]. En effet, lish et al [7] rapportent que 70% des patients bipolaires ont été initialement non diagnostiqués. Le premier diagnostic retenu avant celui du TB est différent selon les études (Tableau 1). Dans l’étude de Suppes [8], l’épisode dépressif majeur(EDM) avait été le plus proposé (44%), suivi de la schizophrénie (34%). Dans l’étude de Hierschfeld [9], l'EDM était retenu dans 60% des cas suivi du trouble anxieux dans 26% des cas. Ghaemi et al [10] rapportent que 40% des patients ont reçu le diagnostic incorrect d'EDM. Nos résultats rejoignent ceux de la littérature puisque 40% de nos patients n'ont pas été diagnostiqués comme bipolaires au début et que le premier diagnostic retenu était celui de l'EDM dans 36% des cas, suivi de l'accès psychotique aigu dans 28% des cas.

**Tableau 1:** Le premier diagnostic retenu avant celui du trouble bipolaire selon les études

	Hierschfeld RM et al (J clin Psy 2003)	Suppes et al (J Affect Disorder 2001)	Notre étude
Episode dépressif majeur	60%	44%	36%
Trouble anxieux	26%		9%
Schizophrénie	18%	34%	16,8%
Trouble de personnalité	18%		10,2%
Abus de substance	17%		
Trouble schizoaffectif	14%		
Trouble psychotique aigu	11%		28%

L’étude de Lish [7] a montré qu'un sujet sur deux a consulté au moins 3 professionnels de santé avant de recevoir un diagnostic approprié. Dans l’étude de Ghaemi [10], 40% des patients avaient été hospitalisés avant de faire le diagnostic du TB. Dans notre étude, 60% des patients ont consulté au moins un psychiatre et 68% des malades ont été hospitalisés au moins une fois avant de recevoir le diagnostic de la bipolarité.

Le retard diagnostic des troubles bipolaires a été rapporté par plusieurs études. Dans une étude clinique multicentrique américaine [5], un délai moyen de 120 mois a été retrouvé. Dans l’étude de lish [7], le diagnostic n'a été posé qu'après 10 ans d’évolution chez un tiers des patients. Dans l’étude de Hierschfeld [9], un délai moyen de 96 mois a été retrouvé. Dansl’étude de Stang [6], le délai moyen du retard diagnostic était de 21 mois mais et presque la moitié (47%) des patients avait un retard de plus de 48 mois. Dans l’étude de Hantouche et al [2], le diagnostic de la bipolarité n'a été fait qu'après cinq ans (60 mois) d’évolution dans presque 40% des cas. Dans notre étude le délai moyen du retard diagnostic est de 64 mois, la moitié des patients avaient un retard de plus de cinq ans.

Il est bien établi que le retard diagnostic du trouble bipolaire a un impact majeur sur le pronostic et l’évolution de la maladie puisque c'est durant cette période que s'installent les principales complications sociales, familiales et professionnelles [1]. Ce retentissement a fait l'objet de plusieurs études. Ainsi selon la DMDA (national depression and manicdepressive association), seulement 37% des patients bipolaires en âge de travailler auraient un emploi aux états unis [7]. Au Royaume uni, 46% des patients bipolaires sont sans travail. Sur le plan familial et affectif, 4 patients bipolaires mariés sur 5, divorcent au moins une fois [3].

Le trouble bipolaire est une pathologie qui met en jeu le pronostic vital, avec un taux de mortalité par suicide atteignant 10 à 15% [1]. Ce taux serait encore plus important pouvant atteindre 15 à 20% chez les patients bipolaires non ou mal pris en charge. D'autre part on estime généralement que 25 à 50% des sujets bipolaires ont fait ou feront une tentative de suicide [3]. Dans notre étude 20,6% des malades avaient fait au moins une tentative de suicide.

## Conclusion

Le retard diagnostic du TB est bien établi. Plusieurs diagnostiques peuvent masquer le trouble conduisant à un retard de prise en charge. Ces données devraient alerter les psychiatres pour favoriser un meilleur dépistage de la manie et de l'hypomanie qui reste les éléments clés du diagnostic de TB, garant d'une instauration de traitements appropriés dans les délais optimaux.
